# Fungal spore diversity reflects substrate-specific deposition challenges

**DOI:** 10.1038/s41598-018-23292-8

**Published:** 2018-03-29

**Authors:** Sara Calhim, Panu Halme, Jens H. Petersen, Thomas Læssøe, Claus Bässler, Jacob Heilmann-Clausen

**Affiliations:** 1 Department of Biological and Environmental Science, University of Jyväskylä, Jyväskylä, Finland; 2 Æbletoften, Nøruplundvej 2, Tirstrup, DK-8400 Ebeltoft, Denmark; 3 Natural History Museum of Denmark/Department of Biology, University of Copenhagen, Copenhagen, Denmark; 4 Centre for Macroecology, Evolution and Climate, Natural History Museum of Denmark, University of Copenhagen, DK-2100 Copenhagen, Denmark; 5 Department of Conservation and Research, Bavarian Forest National Park, Freyunger Str. 2, 94481 Grafenau, Germany

## Abstract

Sexual spores are important for the dispersal and population dynamics of fungi. They show remarkable morphological diversity, but the underlying forces driving spore evolution are poorly known. We investigated whether trophic status and substrate associations are associated with morphology in 787 macrofungal genera. We show that both spore size and ornamentation are associated with trophic specialization, so that large and ornamented spores are more probable in ectomycorrhizal than in saprotrophic genera. This suggests that spore ornamentation facilitates attachment to arthropod vectors, which ectomycorrhizal species may need to reach lower soil layers. Elongated spore shapes are more common in saprotrophic taxa, and genera associated with above ground substrates are more likely to have allantoid (curved elongated) spores, probably to lower the risk of wash out by precipitation. Overall, our results suggest that safe arrival on specific substrates is a more important driver of evolution in spore morphology than dispersal per se.

## Introduction

Dispersal plays a major role in the population dynamics of almost all organisms, and may, depending on the organism, involve mature individuals as well as dedicated dispersal propagules such as pollen, seeds and spores^1,2^. A dispersal event involving dispersal propagules can be divided in liberation, transport and deposition phases^3–6^. Each phase may be affected by both internal factors, i.e. the qualities and the behavior of the dispersal propagule itself, and external factors, i.e. the biotic and abiotic qualities affecting the propagule^2^.

For passive dispersers, the transport and deposition stages of dispersal are mainly driven by external factors including wind, water-currents, precipitation, animal vectors and habitat surface features affecting attachment^4,7,8^. If biotic, these external factors may be subject to co-evolutionary forces, as in the case of fungi associating with specific animal dispersal vectors^9,10^, but in the majority of cases only the dispersal propagules are subject to evolution. Evolution may affect several traits that affect dispersal success during transport and deposition phases, e.g. the size and morphology of the dispersal propagules^11^.

After liberation, dispersal propagules must be able to establish a new reproductive individual, either alone or after fusing with another compatible individual. The initial steps in this process include germination and the establishing of roots or hyphal systems in plants and fungi, respectively^12,13^. The amount of resources available in this step, is one very important factor affecting success in establishment, but the chance of reaching a suitable microhabitat for germination is not less important. The related trade-off between production of many small propagules versus fewer and larger propagules is among the most classical in biology^14^.

In fungi, the basic biology of spore liberation, transport and sedimentation was actively studied already in the early 20^th^ century^3,15,16^, and aquatic fungi with fascinatingly diverse spore morphologies gained particular interest^17,18^. However, for the majority of the fungal kingdom the detailed dispersal biology is still unclear, even though fungi play a major role in the function of all terrestrial ecosystems^19,20^. Only very recently we have gained preliminary knowledge about the large scale connections between the morphology of terrestrial fungal spores and the ecology of the species producing them^21,22^.

Currently it is evident that traits like spore size and shape affect dispersal. Larger spores tend to deposit closer to the source than smaller spores^15,23^, but spore shape does not seem to have a major effect on deposition rate^24^. However, nearly all experimental knowledge considers dry deposition conditions; therefore, basic knowledge about the importance of wet deposition and hydrophobia versus hydrophilia in different taxa is largely lacking^22^. Within agarics (gilled mushrooms), variation in spore shape and size have been shown to have a strong phylogenetic component^25^ whereas spore surface, namely ornamentation, is driven by the trophic status of the species. Indeed, Halbwachs *et al*.^21^ showed that mycorrhizal agarics tend to have ornamented spores much more commonly than saprotrophic species.

To summarize, fungal spores, despite their microscopic size, must not only be able to disperse to new suitable habitat patches, but also to establish vital mycelia therein once these patches are reached. Thus, one can assume that any changes in the ecology of a given fungal taxon may drive strong selective pressures on spore morphology, with effects on liberation, transport and/or deposition success in different niches.

In this paper we investigate the links between spore morphology, trophic status and substrate preference in 781 fungal genera divided across the two major fungal phyla that produce fruit bodies, i.e. *Ascomycota* and *Basidiomycota*. As morphological variables we used spore size, smoothness of spore surface and the four most common basic spore shapes (globose, cylindrical, allantoid and elongated). Two main trophic levels were studied – saprotrophic and ectomycorrhizal fungi, with the former further divided based on substrate specialization (wood-inhabiting, litter decayers and herb stem decayers). Our overall goal was to test if spore traits differ among fungi depending on their trophic strategy, which would reflect an evolutionary impact of niche differentiation. Furthermore, in the case of clear differences in spore traits among trophic guilds, we wanted to interpret these differences in an ecological meaningful way considering the different stages of sexual dispersal in fungi. To pursue our questions we run phylogenetically informed linear models.

## Results

### Differences in spore morphology between saprotrophic and ectomycorrhizal fungal guilds

**Figure 1:**
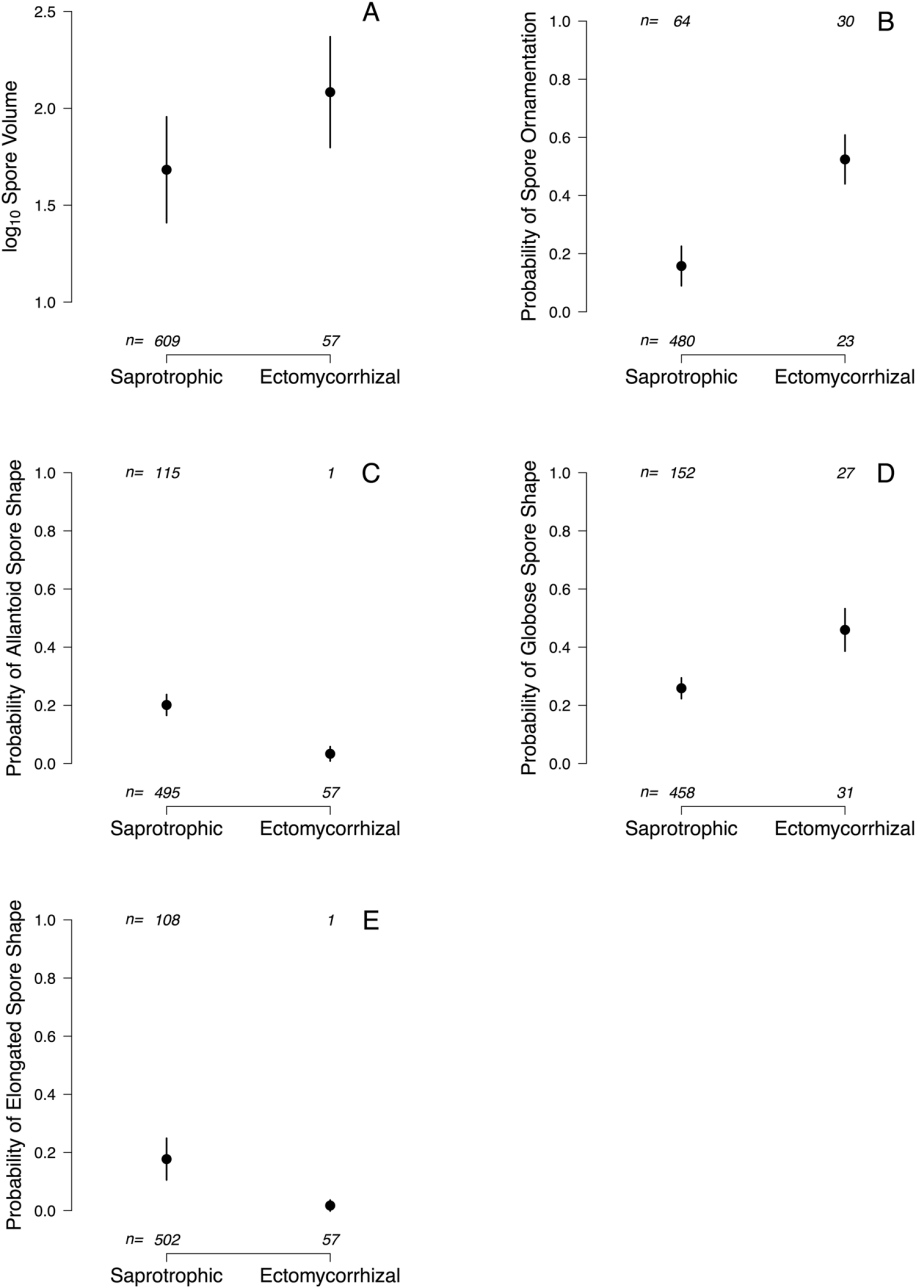
Spore trait differences (estimated mean ± s.e.) across trophic status.

**Figure 2:**
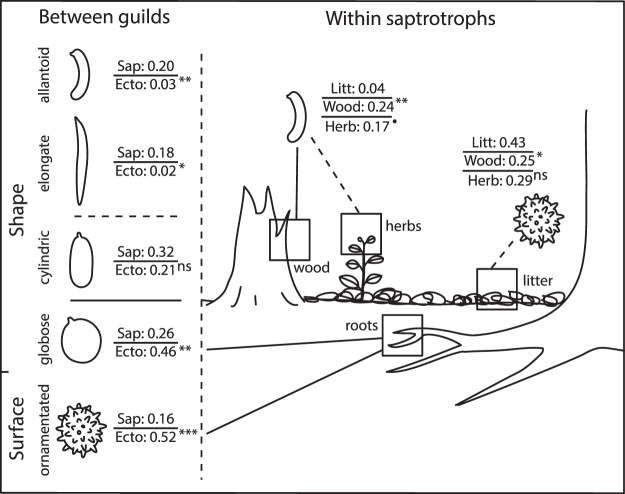
A schematic illustration representing different trophic guilds, substrate associations and spore shapes. Values represent the expected probability of the occurrence of a given feature from logistic linear models controlling for phylogeny. Superscripts refer to significance level for pairwise differences (in the log odds ratios) to the reference level (see Tables 1 and 2).

Ectomycorrhizal taxa had 2.5 times larger spores than saprotrophic species (Table 1; Fig. 1A: volume_sapro_ = 48.2 µm^3^, volume_ecto_ = 121.2 µm^3^). In addition, the odds of ectomycorrhizal taxa having ornamented spores were almost six times larger (i.e. exp(1.77) = 5.87) than for saprotrophic taxa (Table 1; Figs 1B and 2). Also, the odds of ectomycorrhizal taxa having allantoid or elongated spores was only a small fraction (0.14 and 0.08) of the odds for saprotrophic taxa (Table 1; Figs 1C,E and 2). On the other hand, ectomycorrhizal genera were much more likely (i.e. odds 2.44 larger) than saprotrophic taxa to have globose shape (Table 1; Figs 1D and 2). The two trophic levels did not differ in the likelihood of having spores with a cylindrical shape (Table 1; Fig. 2).

**Table 1:** Summary of phylogenetic-controlled models to test for differences in spore traits across trophic status.

	Estimate	s.e.	95% CI	t or z	p
***Log***_***10***_ ***Spore volume***					
λ	0.68		0.53, 0.83		
Intercept	1.68	0.27	1.15, 2.22	6.16	<0.0001
Trophic status	0.40	0.10	0.21, 0.59	4.09	<0.0001
***Ornamentation***					
α	0.28		0.06, 0.51		
Intercept	−1.68	0.51	−2.30, −0.75	−3.27	0.0011
Trophic status	1.77	0.46	1.14, 2.45	3.86	0.0001
***Allantoid shape***					
α	0.63		0.24, 0.74		
Intercept	−1.38	0.22	−0.18, −0.81	−6.16	<0.0001
Trophic status	−1.99	0.77	−3.51, −0.83	−2.58	0.0099
***Globose shape***					
α	0.70^a^		0.29, 0.61		
Intercept	−1.05	0.19	−1.47, −0.59	−5.64	<0.0001
Trophic status	0.89	0.31	0.32, 1.50	2.83	0.0046
***Cylindrical shape***					
α	0.75^b^		0.30, 0.60		
Intercept	−0.77	0.17	−1.10, −0.33	−4.63	<0.0001
Trophic status	−0.57	0.37	−130, −0.03	−1.55	0.1220
***Elongated shape***					
α	0.28		0.13, 0.41		
Intercept	−1.54	0.49	−1.60, −0.83	−3.12	0.0018
Trophic status	−2.51	1.05	−3.14, −1.97	−2.39	0.0167

^a^Bootstrap mean α = 0.41; ^b^bootstrap mean α = 0.41; Note that the coefficients and Wald-type p-values are conditioned on the non-bootstrapped mean estimate for α.

### Differences in spore morphology across saprotrophic substrates

**Figure 3:**
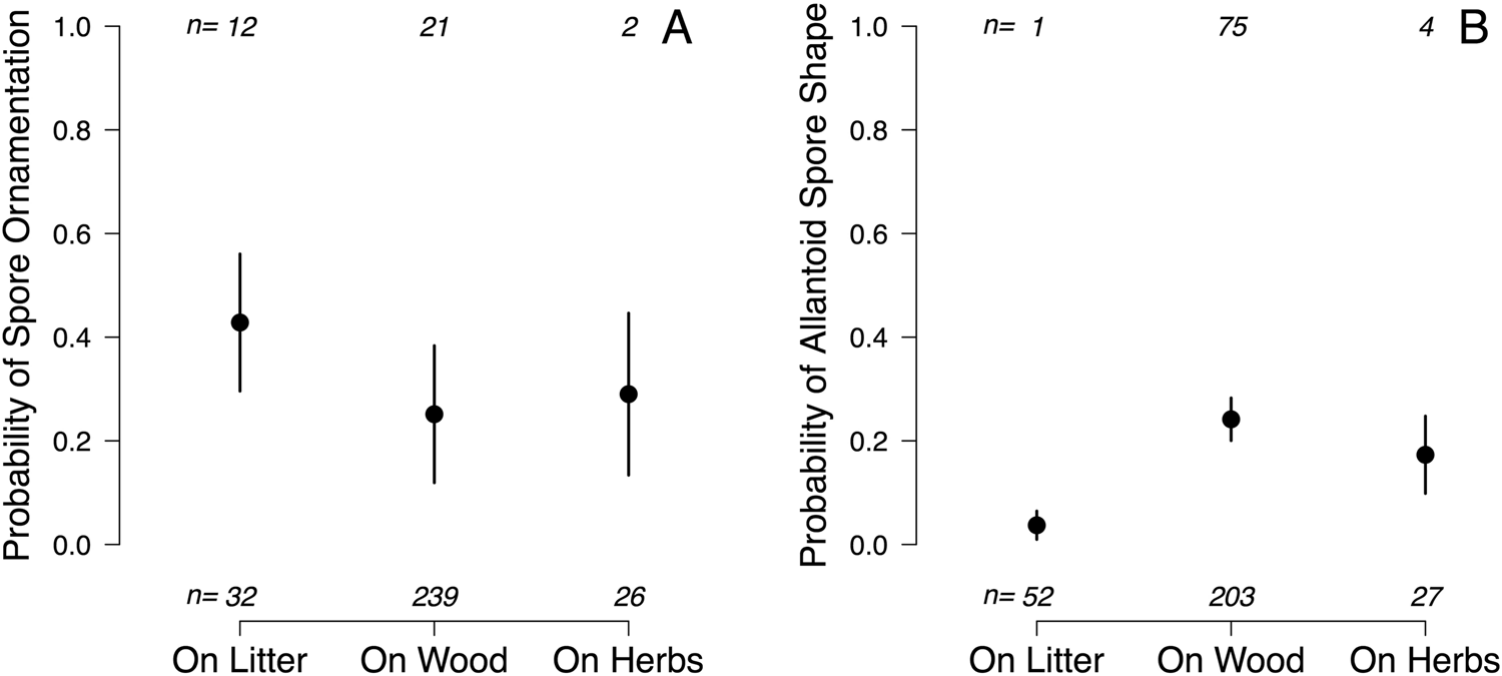
Spore trait differences (estimated mean ± s.e.) across saprotrophic substrates.

No significant differences were found between saprotrophic substrate guilds in spore volume (Table 2; volume_litter_ = 38.4 µm^3^, volume_wood_ = 54.3 µm^3^, volume_herbs_ = 51.3 µm^3^), nor in the occurrence of globose (Table 2; probability of globose spores in litter decayers = 0.21, in wood decayers = 0.26, and in herb decayers = 0.13), cylindrical (Table 2; probability of cylindric spores in litter decayers = 0.32, in wood decayers = 0.31, and in herb decayers = 0.23) or elongated spore shapes (Table 2; probability of elongated spores in litter decayers = 0.17, in wood decayers = 0.19, and in herb decayers = 0.15). In contrast, we found that substrate associations within the saprotrophic guild differed in the odds of having allantoid, and to a lesser degree ornamented, spores. Litter specialists were slightly more likely to have ornamented spores than dead wood specialists (Table 2; Figs 2 and 3A) but the odds of having allantoid spores were considerably higher for wood- (8 times) and herb- (5 times) than for litter-specialists (Table 2; Figs 2 and 3B).

**Table 2:** Summary of phylogenetic-controlled models to test for differences in spore traits across saprotrophic substrate types.

	Estimate	s.e.	95% CI	t or z	p
***Log***_***10***_ ***Spore volume***					
λ	0.52		0.34, 0.71		
Intercept	1.58	0.25	1.10, 2.07	6.38	<0.0001
On wood	0.15	0.10	−0.06, 0.36	1.44	0.1493
On herb stems	0.13	0.15	−0.17, 0.42	0.84	0.3993
***Ornamentation***					
α	0.19		0.09, 0.78		
Intercept	−0.29	0.54	−1.26, 0.82	−0.53	0.5929
On wood	−0.80	0.38	−1.78, −0.20	−2.10	0.0361
On herb stems	−0.61	0.48	−2.06, 0.20	−1.26	0.2062
***Allantoid shape***					
α	0.71		0.16, 0.87		
Intercept	−3.25	0.77	−4.48, −1.88	−4.24	<0.0001
On wood	2.11	0.77	0.87, 3.43	2.76	0.0058
On herb stems	1.69	0.90	−0.07, 3.31	1.89	0.0592
***Globose shape***					
α	0.72^a^		0.20, 0.71		
Intercept	−1.34	0.42	−2.65, −0.43	−3.22	0.0013
On wood	0.28	0.41	−0.26, 1.50	0.69	0.4881
On herb stems	−0.55	0.66	−2.09, 1.07	−0.83	0.4068
***Cylindrical shape***					
α	0.99^b^		0.30, 0.70		
Intercept	−0.74	0.34	−1.57, −0.01	−2.16	0.0310
On wood	−0.06	0.36	−0.58, 0.68	−0.17	0.8649
On herb stems	−0.46	0.55	−1.51, 0.53	−0.83	0.4078
***Elongated shape***					
α	0.21		0.08, 0.56		
Intercept	−1.57	0.76	−3.25, −0.24	−2.08	0.0380
On wood	0.11	0.34	−0.36, 1.65	0.32	0.7482
On herb stems	−0.16	0.51	−1.62, 2.05	−0.32	0.7520

^a^Bootstrap mean α = 0.40; ^b^bootstrap mean α = 0.46; Note that the coefficients and Wald-type p-values are conditioned on the non-bootstrapped mean estimate for α.

## Discussion

Using a comprehensive dataset (n = 787 fungal genera representing the majority of macrofungi known from Europe; cf. Mueller *et al*.^26^), we found clear indications that fungal sexual spore morphology is under strong evolutionary pressure: spore traits showed clear differences between habitat niches, reflecting trophic specialization. It is still largely unknown at what stage in the dispersal process, habitat is most influencial in the evolution of spore morphology. So far, research in spore dispersal in terrestic fungi has mainly focused on spore liberation^11,27^ and dispersal in air^23,28^. In contrast, much less is known on how spores reach suitable spots for establishment (but see^21^), which is a critical feature due to the minimal amounts of stored energy in these microscopic propagules. Below, we try to disentangle these different stages in spore dispersal and provide hypotheses for how habitat associations may drive spore evolution.

### Spore size

We found spore size to reflect trophic strategy, with ectomycorrhizal taxa having larger spores than saprotrophic fungi. The latter is in contrast to Bässler *et al*.^25^, where the same pattern disappeared when analyses were done controlling for phylogeny. The current study’s larger phylogenetic coverage of fungi incorporates a clear difference in the trade-off between spore size and number produced between the two guilds (mycorrhizal and saprotrophic), which suggests adaptations to differences in dispersal and establishment strategies. The surface to volume ratio decreases with increasing spore size, which makes large spores less sensitive to desiccation^25^ and ultraviolet radiation^29^. In line with this, Kauserud *et al*.^30^ found that that fungal species fruiting early in the season (i.e. in the summer) or in areas with continental climate tended to have larger spores than species fruiting later in the season or in more humid climates. In agarics, spore size is linked to fruit body size, so that species with large fruit bodies also tend to have larger spores^31,32^. This suggest a co-adaptation to climatic stress, as large fruit bodies, just like large spores, are better adapted to retain humidity under dry conditions, due to the smaller surface to volume ratio. However, larger fruit bodies also have larger potential for long distance dispersal than smaller fruit bodies due to a higher point of spore release^22^, and since small spores are more likely to disperse over large distances than larger ones^8,23^, the positive correlation between fruit body size and spore size could simply reflect a trade-off between release height and spore size, making larger spores beneficial only in fungi producing large fruit bodies.

Spore size is also important for successful establishment. Larger spores have more resources available for initial hyphal growth, which might be particularly important for mycorrhizal fungi that need to reach suitable host roots^25^. Halbwachs *et al*.^33^ found that spore size increased with resource depletion in mycorrhizal communities, which may suggest an adaptation to prolonged dormancy in spore-banks; the latter has reported been in some ectomycorrhizal fungi^34^.

### Spore shape

A globose spore shape was much more common among mycorrhizal than saprotrophic fungi. It has been shown that these differences also translate into realized community assemblages, indicating the ecological relevancy of this observed pattern^33^. Globose shapes minimize the surface/volume ratio with a given volume and therefore may maximize spore survival in harsh conditions. However spore shape also affects spore liberation. In the ascomycetes, where spores are discharged explosively from asci, cylindrical shapes maximize the flight speed of liberated spores^11^. In basidiomycetes, spore shape affects the size of the so-called Buller´s drop, which determines the force with which spores are released from basidia^27^. Although the optimal shape of a spore is affected by several selection forces, our results strongly indicate that many ectomycorrhizal fungi sacrifice the adaptation of optimal liberation of their spores to achieve globose shapes, supporting that they have higher needs for energy resources for primary mycelial growth or dormancy survival in order to successfully infect plant roots.

Despite the majority of both ectomycorrhizal and saprotrophic fungi having rather roundish, globose to ellipsoid-cylindrical spore shapes, the latter guild showed a large number of genera with different kinds of elongated spore shapes, either sausage shaped (allantoid), straight or irregularly curved. These shapes were almost totally absent among mycorrhizal genera (we recorded only one mycorrhizal genus with allantoid, and one with elongated spores). Allantoid spores even showed significant associations within the saprotrophic guilds, being significantly more common among wood and herb stem specialists compared to litter decayers.

Hussein *et al*.^24^ showed that basidiomycete spore shape does not affect flight capacity when spore size is controlled for. Therefore, our results suggest that the deviating spore shapes reflect differences in deposition strategies across species. Ingold^3^ distinguished three types of spore deposition in terrestrial environments, i.e. sedimentation (passive deposition due to gravity), impaction (by force of wind) and wash-out by rain. These processes are affected by spore size and shape in complex and poorly understood ways^8,24,35,36^, but the general trends found in our study suggest that allantoid shapes are advantageous for deposition on above ground substrates (wood and herb stems), while roundish spores are advantageous for reaching the litter layer of the forest floor (or the plant roots hidden within it).

Very little is known about the meaning of curviness of spores, as represented especially in the allantoid class. Studies on impaction involving curvy, sigmoid spores of aquatic fungi have shown that curviness may have to do with reaching the right position at the exact moment when the spore touches a surface^37^. Indeed, a curved shape may always force the spore to reach the very same position when attaching to the surface, thus enabling immediate germ tube growth from a designated germ pore or slit in the spore-wall^37^. Based on our results we suggest that wood- and herb-inhabiting fungi may have allantoid spores to assist the attachment on vertical or otherwise challenging exposed surfaces under the impact of both impaction and wash-out. The latter remains to be tested in laboratory conditions in order to contrast the latter with alternative explanations, such as that allantoid spore shape are associated with particular animal vectors or that this shape allows direct passage through open stomata or lenticels in living plants. Many herb and wood-inhabiting fungi infect plants while these are still alive, but only turn into active decomposers after plant dead or senescence^38,39^.

### Ornamentation

Ornamentation was much more common in mycorrhizal than saprotrophic genera, suggesting ornamentation to be a very beneficial trait for mycorrhizal fungi, as also demonstrated by Halbwachs *et al*.^21^ for agarics. Ectomycorrhizal fungi need to germinate adjacent to rootlets, typically in subsurface humus layers, and there is some evidence that microarthropods are dispersing ornamented spores effectively to adequate spots for germination in the soil^40,41^. A similar phenomenon exists with moss pollen^42^, and on a scale magnitudes larger, with plant seeds^43^. It may well be that for mycorrhizal fungi passive dispersal is only rarely capable to bring the spore close enough to the root tips of potential hosts, and therefore animal dispersal agents are needed. At least it seems difficult to believe that ornamentation could facilitate passive dispersal by water through the soil column, the importance of which is anyway debatable^44^.

For saprotrophic fungi the potential substrates are usually present directly on, or close to the surface where spore may land based on passive forces. The connection to animal-assisted dispersal is further supported by the fact that among saprotrophic genera, ornamentation was more common among the litter decayers than in genera utilizing above ground substrates. It may well be that some litter decayers also benefit from soil arthropods taking them to deeper litter layers away from surface hazards such as desiccation or ultraviolet radiation^29^.

## Conclusions

Recent discussions on dispersal of windborne spores have largely focused on the “how to fly” -part of the dispersal process. Despite the massive numbers of spores released in many fungi^45,46^ challenges related to deposition and germination may be more important for successful dispersal than challenges in transportation. Overall our study shows that spores of fungi belonging to different trophic guilds differ in morphology, potentially because they need to reach different substrates and stay there to allow germination, as investigated mainly among aquatic fungi decades ago (reviewed by Jones^47^).

Obviously, spores of mycorrhizal fungi must reach root tips deep down in the debris, and hence they may benefit from arthropod vectors to assist in the last transport step, which may be facilitated by ornamentation. Spores of litter decaying fungi should avoid deposition on above ground surfaces but generally do not need to go deeper after they have reached the forest floor. Thus a roundish shape with smooth surface may be the most advantageous. And finally, spores of fungi that decay above ground substrates, including wood and herbs must have some qualities helping them to attach to a surface whenever they touch one to reduce the risk of getting lost with the wind or water. The allantoid shape could be advantageous in that respect. Our study is the most comprehensive so far on spore morphology in fungi, and despite its intrinsic exploratory nature, we find that it provides an important basis for hypothesis-driven future research, involving both field studies and experimental work. We hope that our focus on the ecological scenarios at the ‘touch-down’ stage will inspire similar research also in other systems, where dispersal propagules are microscopic and morphologically diverse.

Due to the use of genus level data our study was focused on very general trends. We have not explored infra-generic variation in spore morphology, and have deliberately omitted genera encompassing different ecological strategies. However, follow-up studies on clades with huge variation in spore morphology and trophic roles would be valuable to deepen our understanding of the ecological signal in the evolution of spore morphology in fungi. Candidate clades for such studies include the genera *Ramaria* s.lato^48^ (*Basidiomycota*) and *Peziza* (*Ascomycota*)^49^ as well as the family *Entolomataceae* (*Basidiomycota*)^50^. In the same line, it is worth emphasizing that we considered only sexual spores, whereas in reality many fungi disperse also, in some cases almost exclusively, by asexual spores. Many classic genera of asexual fungi defined based on spore morphology and ontogenesis, have been shown to be highly polyphyletic when tested with molecular data^51^, suggesting strong convergent evolution. At least in aquatic fungi, evolution of conidial morphology has been shown to be dynamic reflecting ecological specialization rather than taxonomy^52^, but so far similar studies on morphological evolution in air-dispersed conidia are to our knowledge lacking.

## Material and Methods

### Data collection

The data used in this paper were obtained from the extensive database compiled for the Mycokey 4.0TM fungal identification software^53^. The database is compiled at the genus level and is based on extensive literature searches on the included genera. It contains ecological and morphological data on all fruit body forming basidiomycetes and apothecia forming ascomycete genera occurring in northern Europe (i.e. Europe north of the Alps). We used a subset of these data comprising n = 781 fungal genera, for which morphometric data, phylogenetic classification and ecological information was most reliable and/or comprehensive. One third of the genera (n = 286 genera) belong to Ascomycota and the rest (n = 495 genera) to Basidiomycota.

Spore morphology was assessed as overall size, surface ornamentation and shape category. Spore size was measured as spore volume by following the equation for volume of an ellipsoid [4/3*π*(length/2)*(width/2)^2^]. Minimum values in MycoKey were used in the analysis, but the same qualitative results were found with maximum and average dimensions. Spore ornamentation was classified in an exclusive manner, where only genera consistently recorded as having a smooth or an ornamented spore surface were included. Only a small percentage of genera (10%, n = 78) have variation in this trait. Four main categories from Mycokey were used to classify spore shape: globose-subglobose (hereafter globose), cylindrical, allantoid and elongated (which includes vermiform, filiform, sinuous, cucumber, boomerang and stocking-shaped, see Fig. 2 for example drawings of different shapes). Spore shape traits were scored in an inclusive manner, since there is often considerable variation in spore shape within genera: only 42% of taxa (n = 331) were homogeneous in spore shape classification.

Trophic status was classified in an exclusive manner, i.e. only genera that were homogenous in this trait, both for the broader – saprotrophic vs. ectomycorrhizal – or the narrower – within saprotrophic guild substrate association (litter, wood or herb stems) – classification, were included. Rarer substrates were omitted for the current analyses. Only a small percentage of genera (5%, n = 38) were classified as heterogeneous in trophic status. The saprotrophic guild was notably more common than mycorrhizal, with n = 611 and n = 58 genera, respectively. The great majority of saprotrophic taxa were wood-inhabiting (n = 278 genera) followed by litter-inhabiting (n = 53 genera) and herb inhabiting (n = 31 genera).

### Phylogeny compilation

We compiled a topology based fungal phylogeny based on Hibbett *et al*.^54^ for higher node relationships, and resolved nodes down to the genus level using maximum likelihood trees from other publications. Genera that were missing in published phylogenies were placed in polytomies at the lowest known taxonomic grouping (using Mycokey taxonomic information). A total of n = 781 genera were included in these analysis. Branch lengths were standardized at one, except for those reflecting ‘soft’ polytomies. These polytomic nodes were randomly resolved into dichotomic nodes using the function ‘multi2di’ from the package ‘ape’^55^ in R version 3.2.2 (R Core Team 2015). The ‘new node’ branch lengths were set at zero, since these polytomies are considered to be ‘soft’.

### Statistical Analyses

All statistical analyses were done in R version 3.2.2 (R Core Team 2015). The function ‘gls’ from the package ‘nlme’^56^ was used to run phylogenetically-controlled models, where spore size (log_10_-transformed spore volume) was the dependent variable. We used logarithm of base ten transformation in order to account for the incredible range of volume values (~10^5^ fold), which were recorded for the subphylum Pezizomycotina. Following Symonds and Blomberg^57^ online material, phylogenetic non-independence was accounted for by setting the correlation structure argument of ‘gls’ as ‘corPagel’ from the package ‘ape’^55^. It also then provides an estimate of λ, the phylogenetic signal in the residuals. Lambda is a measure between 0 (no phylogenetic effect i.e. independence) and 1 (pattern explained by Brownian motion phylogenetic relationships)^58,59^. Using the function ‘intervals’ from ‘nlme’ package, we obtained 95% confidence intervals for lambda as well as the fixed effect parameters. We have also compared the fit of our models where lambda was estimated with models, where lambda was set at 0 or at 1.

Spore ornamentation and spore shape categories were coded as binary response variables. Therefore, we used phylogenetically-controlled logistic regressions using the function ‘phyloglm’ from the package ‘phylolm’^60^, and following instructions in Ives and Garland^61^. The method ‘logistic_IG10′^62^ was used in all but one model due to non-convergence. In that case, the method ‘logistic_MPLE’ was used and several starting points of the parameter alpha (the phylogenetic correlation) were used in order to insure a global (cf. local) maximum likelihood estimates (Lam Ho, online pers. comm.). The model with the maximum (penalized) likelihood was selected. By running ‘phyloglm’ with bootstrap of n = 1000, we obtained the 95% confidence intervals for both phylogenetic and fixed effects parameters. The phylogenetic signal α estimated by ‘phyloglm’ can be interpreted as weak when α ≈ 3, moderately strong when α ≈ 1 and very strong when α ≈ 0.35^61,62^. As with lambda, weak signals in α reflect low phylogenetic dependence of the relationship between dependent and independent variables.

Models were fitted to compare spore size, the occurrence of spore ornamentation and of each of the four shape categories described above between saprotrophic and mycorrhizal genera. The same was done for the comparison across the three substrates of saprotophic taxa (litter, wood and herb stems). Both ‘gls’ and ‘phyloglm’ model objects outputs provide the information needed to calculate (back-transformed) mean estimates and standard errors for all the levels of categorical predictors (cf. just the intercept). We used the delta method as described in the online tutorial by UCLA Statistical Consulting Group (http://www.ats.ucla.edu/stat/r/faq/deltamethod.htm) to obtain standard error values. These were used in figure plotting.

A fully annotated R script, fungal trait data and phylogeny used in the analyses are given as electronic appendices (1, 2a, 2b and 3, respectively).
